# Maria Teresa Carrì Obituary

**DOI:** 10.1038/s41419-018-1193-6

**Published:** 2018-11-19

**Authors:** Giuseppe Rotilio

**Affiliations:** University of Tor Vergata, Dept Biochimica, Rome, Italy


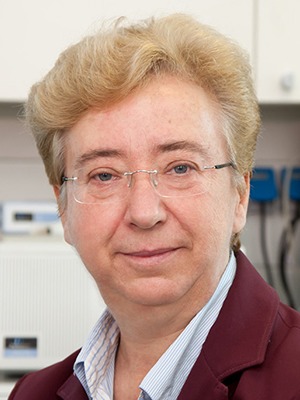


On July 26th, Maria Teresa Carrì prematurely left us after a short battle with an incurable disease. Presently acting as Full Professor of Biochemistry at the University of Rome Tor Vergata, she did not initially train as a Biochemist, completing a Biology degree in 1981. I recruited Maria as a young post doc 3 years later, after which she moved to my laboratory at the Department of Biology at the newly established Second University of Rome, now University of Rome Tor Vergata. This decision was part of a larger plan, as by that time Rome was thriving as a dynamic Centre of Biochemistry research. Indeed, at the historical Institute of Biological Chemistry at the University of Rome La Sapienza, an internationally renowned group of experts were leading research in the fields of enzymology and protein structure-function relationships. Their approach was substantially that of Chemistry, supported by a pioneering expertise in the most advanced techniques of spectroscopy, fast-kinetics and physico-chemical analysis of macromolecules. The opportunity to build a new distinct laboratory elsewhere in Rome therefore seemed to me to be a unique opportunity to complement this work through the use of recombinant DNA and cell culture technologies. Maria Teresa was indeed an early expert in these methodologies owing to experiences gained as a part of her doctoral thesis in an innovative laboratory of Cell and Molecular Biology at the Institute of Histology of the Faculty of Sciences, University La Sapienza, and the subsequent period spent as EMBO fellow at the Basel Biozentrum.

The focus of my research in the new University was still on Cu,Zn-Superoxide dismutase (SOD 1), a protein that I had extensively studied at La Sapienza University with a focus on the structure of its metal-binding sites and its mechanism of catalytic action. Here, Maria assembled and developed a new laboratory that sought to exploit her knowledge on molecular biology and finally led a specific project that aligned with the interests the biochemical section of the Tor Vergata Department of Biology. Here, indeed, my coworkers sought to understand the central role of oxygen and energy metabolism, encompassing different areas of cell biology. Maria Teresa’s task, in the light of our competence and facilities in that period, was challenging in many respects. She engaged in *SOD 1* gene manipulation not only for cloning and expressing SOD enzymes with mutated activities and structures to enhance our understanding its extraordinary catalytic efficiency, but also the transfection of such variants and subsequent detection of altered molecular functions. As a matter of fact, even now, the biological role of this protein is still largely a mystery, as enzymatic dismutation of superoxide radicals appears to be a redundant process, or even potentially toxic because of the hydrogen peroxide produced. A possible hint came in 1993 when it was reported that a significant number of patients affected by the familial form of amyotrophic lateral sclerosis (ALS), a lethal motor neuron degenerative disease, carried-specific SOD 1 mutations. Soon after, we started a project on ALS-linked SOD mutants. This included purification, molecular analysis and spectroscopy, whilst Maria Teresa developed an original cell model to identify, if any, the neurodegenerative effects of inserting ALS-linked SOD mutants in neuroblastoma cells. This became her predominant interest for many years, and developed into a leading role amongst the community of scientists working on molecular mechanisms and pathophysiology of ALS and neurodegeneration.

As early as 1997 she pointed out the key role of mitochondria in the molecular mechanisms of ALS pathogenesis, by discovering effects of intracellular calcium mishandling in transfected neuronal cells. This was a novel and original standpoint, a significant precursor to the major topic of molecular ALS research we have today. The next step she took was moving to animal models in order to obtain evidence in support of her pioneering work carried out in 2003, proposing that ALS is to be viewed as a multifactorial disease with not only motor neuron cells contributing to establish the pathological phenotype, but rather their cross-talk with other cell types. This idea was obviously of great relevance for the identification of possible targets in ALS drug therapy and led to Maria Teresa broadening her involvement to ALS clinical treatment as well. The importance of her recent research activity on these newly elucidated aspects of ALS pathogenesis was highlighted by publications of these findings in high rank journals, including *Cell Death and Disease*. In fact, she left us just at the dawn of a day predictably full of important innovative findings. *Is cadit ante senem qui sapit ante diem*.

Needless to say, Maria Teresa was a very active promoter of ALS research at both national and international levels through her very appreciated membership of funding committees, expert panels, society scientific boards and through editorial roles for neuroscience journals. In 30 years of personal acquaintance with her as a mentor, colleague and friend I admired her calm determination in planning and deciding and her boundless energy and enthusiasm in fulfilling her scientific goals. No doubt her great success as a leader of younger scientists was also due to her civil approach and respect for others, and indeed the loss of her human qualities is what we feel we will miss the most.

Giuseppe Rotilio M.D., Ph.D.

Professor of Biochemistry

